# Artificial intelligence for older people receiving long-term care: a systematic review of acceptability and effectiveness studies

**DOI:** 10.1016/S2666-7568(22)00034-4

**Published:** 2022-04

**Authors:** Kate Loveys, Matthew Prina, Chloe Axford, Òscar Ristol Domènec, William Weng, Elizabeth Broadbent, Sameer Pujari, Hyobum Jang, Zee A Han, Jotheeswaran Amuthavalli Thiyagarajan

**Affiliations:** a Department of Psychological Medicine, University of Auckland, Auckland, New Zealand; b Department of Health Services and Population Research, Institute of Psychiatry, Psychology and Neuroscience, King's College London, London, UK; c Stanford Prevention Research Center, Department of Medicine, Stanford University, Stanford, CA, USA; d Department of Digital Health, World Health Organization, Geneva, Switzerland; e Department of Maternal, Newborn, Child and Adolescent Health and Ageing, World Health Organization, Geneva, Switzerland; f WHO/ITU Focus Group on Artificial Intelligence for Health (FG-AI4H), Geneva, Switzerland

## Abstract

Artificial intelligence (AI)-enhanced interventions show promise for improving the delivery of long-term care (LTC) services for older people. However, the research field is developmental and has yet to be systematically synthesised. This systematic review aimed to synthesise the literature on the acceptability and effectiveness of AI-enhanced interventions for older people receiving LTC services. We conducted a systematic search that identified 2720 records from Embase, Ovid, Global Health, PsycINFO, and Web of Science. 31 articles were included in the review that evaluated AI-enhanced social robots (n=22), environmental sensors (n=6), and wearable sensors (n=5) with older people receiving LTC services across 15 controlled and 14 non-controlled trials in high-income countries. Risk of bias was evaluated using the RoB 2, RoB 2 CRT, and ROBINS-I tools. Overall, AI-enhanced interventions were found to be somewhat acceptable to users with mixed evidence for their effectiveness across different health outcomes. The included studies were found to have high risk of bias which reduced confidence in the results. AI-enhanced interventions are promising innovations that could reshape the landscape of LTC globally. However, more trials are required to support their widespread implementation. Pathways are needed to support more high-quality trials, including in low-income and middle-income countries.

## Introduction

To meet the goals of the UN Decade of Healthy Ageing, it is necessary that countries meet the health-care needs of current and future older populations through innovative solutions. According to WHO, long-term care (LTC) services are defined as: “Services to ensure that people with or at risk of significant loss of physical and mental capacity can maintain a level of functional ability consistent with their basic rights, fundamental freedoms, and human dignity…Services are provided by both unpaid caregivers (typically family but also volunteers) and paid care staff…‘Long-term care services’ covers care at home, in the community and in facilities (residential long-term care facilities, nursing homes or other group living facilities).”^1^ LTC services typically assist people with activities of daily living, and encourage social participation and management of chronic health conditions.^1^, ^2^

In recent years, artificial intelligence (AI) has begun to reshape the global landscape of LTC. AI refers to systems that analyse their environments and take actions to achieve specific goals with a degree of autonomy.^3^, ^4^, ^5^ AI can be based in software systems and act in virtual spaces (eg, conversational agents, facial recognition systems), or be based in hardware and act in physical environments (eg, robots). AI techniques include machine learning (eg, neural networks, deep learning), computer vision (eg, image classification, object tracking), pattern detection, and natural language processing, among others. AI-enhanced interventions refer to technology interventions that include an AI component (eg, environmental sensors with classification algorithms for fall detection).

Increasingly, AI-enhanced interventions have been developed to support the health and capacity of older people receiving LTC, aiming to expand the reach of care provision and its efficiency, and reduce caregiver burden.^6^ These technologies have the potential to improve workforce sustainability (eg, acting as additive support to caregivers), to address service inequity (eg, through providing services in remote areas where LTC availability is low and demand is high), and to increase the efficiency of information systems and data analysis of people in need of LTC. However, the research field is developmental.

The literature on AI-enhanced interventions in LTC services has yet to be systematically synthesised and assessed for quality. Existing reviews have focused on different types of smart technologies such as social robots and environmental sensors for assisting older people, albeit not focused in LTC specifically, which might affect the generalisability of results and clinical implications.^7^, ^8^ Other reviews have focused on specific robots in elderly care facilities (eg, PARO) which limits comparison with other AI-enhanced technologies that might be more affordable and accessible.^9^, ^10^

To address this gap, we systematically reviewed the literature on the acceptability and effectiveness of AI-enhanced interventions for older people receiving LTC. This review aims to address the following research questions: (1) What AI-enhanced interventions have been trialled in LTC services? (2) What AI-enhanced interventions have been shown to be effective for older people receiving LTC? (3) What AI-enhanced interventions have been shown to be acceptable to older people receiving LTC?

## Methods

### Search strategy and selection criteria

A preregistered protocol for this review is available on PROSPERO (registration number: CRD42020218154). The review has been prepared following the Preferred Reporting Items for Systematic Reviews and Meta-Analyses (PRISMA) guidelines (for checklist, see appendix pp 1–3). No protocol deviations occurred aside from an updated search strategy which improved the specificity of results.

A systematic search was conducted on Embase, Ovid, Global Health, PsycINFO, and Web of Science. Searches were limited to title, abstract, and keywords. No restrictions were placed on publication dates. Systematic searches were conducted on Jan 22, 2021. The search terms are presented in the appendix (pp 4–7). Manual searches were conducted for relevant publications of included authors and reference lists of relevant systematic reviews.

The eligibility criteria are presented in the appendix (pp 8–10). Studies were screened for inclusion in two stages (abstract-only screen, full-text review) with training beforehand. An abstract-only screen was conducted using Rayyan software. One rater (WW) screened all search results and three raters (CA, KL, ORD) each screened one-third in duplicate. After abstracts were screened, raters met to resolve disputes and derive a final list of papers for full-text review. A full-text review was conducted using Covidence software. Two independent raters (KL, WW) examined full-text articles against the eligibility criteria. When unclear if an intervention included AI, raters contacted the authors and searched for additional information (eg, peer-reviewed articles on the intervention, developer websites). This process was followed for all technologies, including social robots. Raters achieved moderate agreement in the initial ratings (*k*=0·45). Raters met to achieve consensus on a final list of included papers alongside two members of the research team (CA, ORD).

### Data analysis

Two independent raters (CA, ORD) extracted the data in duplicate into separate spreadsheet forms (available upon request to the corresponding author). Data were extracted on study details (eg, institution, publication year, country, setting), study design, population (eg, demographic and health characteristics, inclusion and exclusion criteria, sample size, group differences), intervention (technology, length of exposure), comparators, outcomes (measure, assessment timing), and results (mean scores, effect sizes, p values). Raters met at the end of data extraction to resolve discrepancies.

A risk of bias evaluation was conducted independently in duplicate by two trained raters (CA, ORD) using the Revised Cochrane Risk of Bias tool for randomised trials (RoB 2), Revised Cochrane Risk of Bias tool for cluster-randomised trials (RoB 2 CRT), and the Risk of Bias in Non-randomised Studies of Interventions (ROBINS-I) tool for non-randomised trials.^11^ Risk of bias was evaluated at the study level to assess the internal validity of included studies. Raters met with a third member of the research team to resolve disputes (MP).

A narrative synthesis was conducted due to the high heterogeneity of outcomes and measures across studies, using guidance by Popay and colleagues.^12^ Study characteristics were tabulated and presented narratively. Results were presented and stratified according to whether the study had a control group. Health outcomes that were assessed in more than three papers for each type of study design were tabulated. The overall findings were reported in a narrative format.

## Results

**Figure 1:**
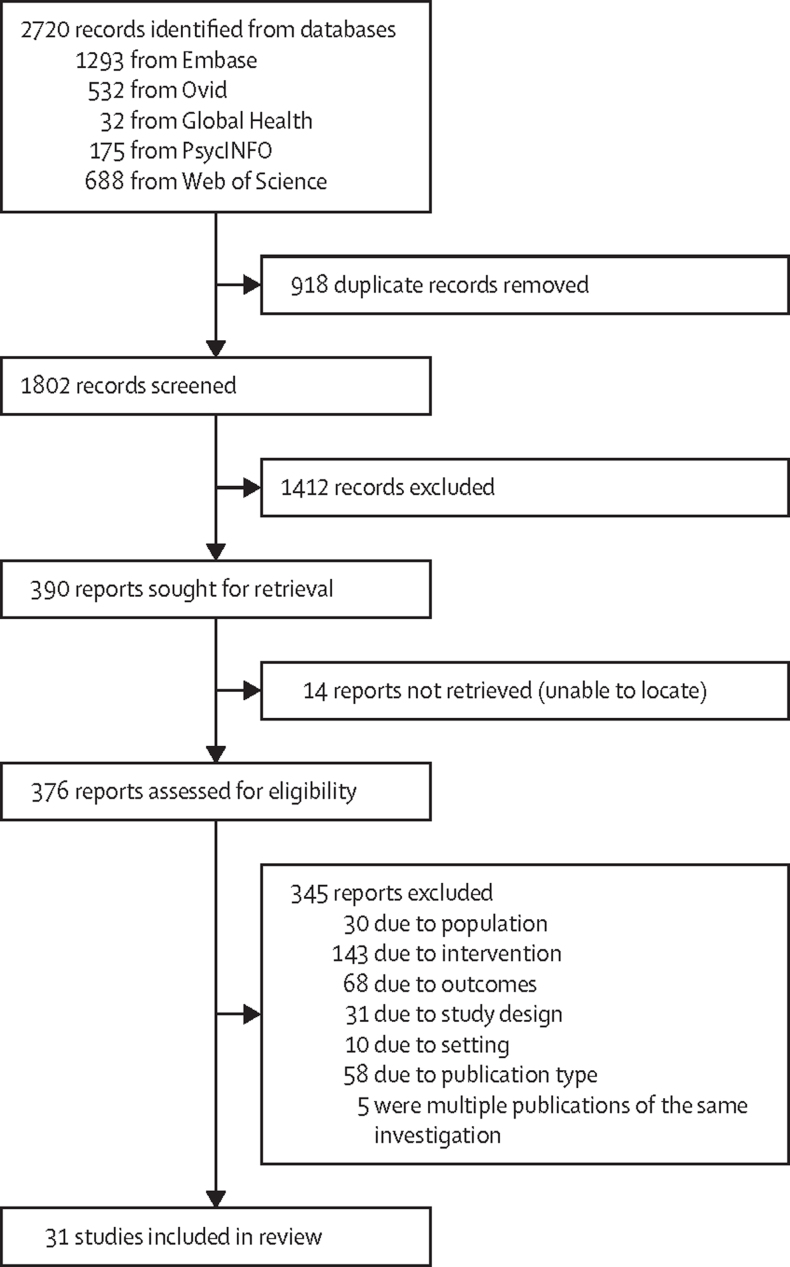
PRISMA flowchart

The literature search identified 2720 records of which 31 were eligible for review (figure 1).

Table 1 reports the sociodemographic characteristics of the included studies.^13^, ^14^, ^15^, ^16^, ^17^, ^18^, ^19^, ^20^, ^21^, ^22^, ^23^, ^24^, ^25^, ^26^, ^27^, ^28^, ^29^, ^30^, ^31^, ^32^, ^33^, ^34^, ^35^, ^36^, ^37^, ^38^, ^39^, ^40^, ^41^, ^42^, ^43^ There were 15 controlled trials and 14 non-controlled trials. All studies were conducted in high-income countries, with the majority from North America, Australasia, and Europe.

**Table 1:** Characteristics of included studies

	**Intervention type**	**Country, study year**	**N**	**Setting**	**Age; sex (%)**	**Trial design**	**Intervention and control**	**Length of exposure**	**Outcomes**	**Timepoints measured**
**Controlled trials**										
Banks et al (2008)^13^	Robot	USA, year not reported	38	Three LTC facilities	Age: not reported; sex % not reported	RCT	Intervention: social robot (AIBO); control: no social robot or living dog	30 min, once a week for 8 weeks	Loneliness (UCLA loneliness scale); attachment (MLAPS)	T1: baseline; T2: week 7
Broadbent et al (2016)^14^	Robot	New Zealand, 2011–12	53 residents 53 staff	Rest and nursing homes	Mean age: 85·5 years; female: 77%	Non-randomised controlled trial	Intervention: social robots (Guide and Cafero); control: standard care	Robots left on 24/7 for 12 weeks in total	Acceptability (resident); depression (GDS); quality of life (resident and staff); dependency (resident)	T1: baseline; T2: week 12
Cohen et al (2016)^15^	Wearable	Switzerland, 2014–15	34	Home care	Mean age: 83·2 years; female: 67%	Pilot RCT	Intervention: intelligent wireless sensor system; control: standard care	13 weeks	Acceptability (participants and caregivers)	T1: 2 weeks before baseline; T2: week 12
Jøranson et al (2015, 2016)^16^, ^17^	Robot	Norway, 2013–14	53	Nursing homes, participants with dementia	Mean age: 84 years; female 67%	Cluster RCT	Intervention: social robot (PARO); control: standard care	30 min, twice a week for 12 weeks	Agitation (BARS); depression (CSDD); quality of life (QUALID)	T1: baseline; T2: week 12; T3: week 25
Liang et al (2017)^18^	Robot	New Zealand, year not reported	30 dyads (LTC consumers and caregivers)	Dementia day care centre and homes, participants with dementia	Age range: 67–98 years; female: 64%	Pilot RCT	Intervention: social robot (PARO); control: standard care	1 hour, two to three times a week for 12 weeks	Agitation (CMAI-SF); facial expressions; social interactions; cognition (ACE); neuropsychiatric symptomatology (NPI-Q); depression (CSDD); medication usage; blood pressure; heart rate; hair cortisol	T1: baseline; T2: week 6; T3: week 12
Libin et al (2004)^19^	Robot	USA, year not reported	9	Nursing home	Mean age: 90 years; female: 100%	Pilot trial	Intervention: social robot (NeCoRo); comparator: plush toy cat	10 min, two sessions—one with robot, one with toy	Agitation (ABMI); affect (LMBS); cognition (Global Deterioration Scale)	T1: baseline; T2: after session
Moyle et al (2013)^20^	Robot	Australia, 2011	18	LTC facility	Mean age: 85·3 years; female: year not reported	Pilot crossover RCT	Intervention: social robot (PARO); control: reading control group	45 min, three times a week for 5 weeks	Wandering (AWS); quality of life (QOL-AD); apathy (AES); depression (GDS); anxiety (RAID); emotions (OERS)	T1: baseline; T2: week 5
Moyle et al (2017, 2018)^21^, ^22^	Robot	Australia, 2014–15	415	28 LTC facilities	Mean age: 84·1 years; female: 7·4%	Cluster RCT (three arms)	Intervention: social robot (PARO); control: standard care and plush toy	15 min, three times a week for 10 weeks	Engagement; mood; agitation (CMAI-SF); motor activity; sleep activity; qualitative	T1: baseline; T2: week 5; T3: week 10
Pu et al (2020)^23^	Robot	Australia, 2018–19	43	Three LTC facilities, participants with dementia	Mean age: 86·0 years; female: 70·7%	Pilot RCT	Intervention: social robot (PARO); control: standard care	30 min daily for 6 weeks	Motor activity; sleep; depression (CSDD); anxiety (RAID); agitation (CMAI-SF); pain (PAINAD); qualitative	T1: baseline; T2: week 6
Rantz et al (2017)^24^	Environmental Sensors	USA, year not reported	171	13 assisted living communities	Mean age: 84·8 years; female: 73·6%	Controlled trial	Intervention: environmentally embedded sensors; control: standard care	24/7 for 1 year	Gait (GAITRite*); short physical performance battery (SPPB); hospitalisation; activities of daily living (ADLs and IADLs); depression (GDS); cognition (MMSE)	T1: baseline; T2: month 4; T3: month 8; T4: month 12
Rantz et al (2012)^25^	Environmental sensors	USA, year not reported	41	LTC facility	Mean age: 84·5 years; female: 66%	Non-randomised controlled trial	Intervention: environmental sensors (SMARTA); control: no sensor	1 year	Cognition (MMSE); depression (GDS); gait (GAITRite*); balance; qualitative	T1: baseline; T2: month 4; T3: month 8; T4: month 12
Robinson et al (2013)^26^	Robot	New Zealand, year not reported	34	Hospital and rest home care facility	Age range: 55–100 years; sex % not reported	RCT	Intervention: social robot (PARO); control: standard care	1 hour, twice a week for 12 weeks total	Loneliness (UCLA loneliness scale); depression (GDS); quality of life (QOL-AD)	T1: baseline; T2: week 12
Thodberg et al (2016)^27^	Robot	Denmark, 2016	100	Four nursing homes	Median age: 85·5 years; female: 69%	RCT (three arms)	Intervention: social robot (PARO); control: normal activities or toy cat	10 min, twice a week for 6 weeks total	Physical contact; eye contact; verbal communication	T1: baseline; T2: week 2; T3: week 4; T4: week 6
Valenti- Soler et al (2015)^28^	Robot	Spain, 2012–13	121	Nursing homes and day care, participants with dementia	Mean age: 83·5 years; female: 81·5%	RCT (three arms)	Intervention: social robot (PARO or NAO); control: normal activities	30 min, twice a week for 3 months	Cognition (Global Deterioration Scale); cognition (MMSE); neuropsychiatric symptomatology (NPI); apathy (APADEM-NH and AI); quality of life (QUALID)	T1: baseline; T2: 3 months
Wilmink et al (2020)^29^	Wearable sensors	USA, year not reported	490	Six assisted living communities	Mean age: 88·1 years; female: 69·2%	Restrospective study	Intervention: wearable sensors (CarePredict); comparator: no sensor	1 year	Hospitalisation; falls	T1: baseline; T2: year 1; T3: year 2
**Non-controlled trials**										
Barrett et al (2019)^30^	Robot	Ireland, year not reported	10	Nursing homes, participants with dementia	Mean age: 83 years; female: 70%	Pre-post	Intervention: social robot (MARIO)	60 min, three times a week for 4 weeks	Acceptability; accessibility; depression (CSDD); quality of life (QOL-AD)	T1: baseline; T2: week 4
Bemelmans et al (2015)^31^	Robot	Netherlands, 2012–13	91	Six LTC facilities, participants with dementia	Age: ≥65 years; female: 80%	Quasi experimental time series ABAB	A: standard care; B: social robot (PARO)	15 min, 1 month for each phase (PARO used five times in each B phase)	Individually Prioritized Problems Assessment (IPPA); mood assessment; GIP-28	T1: baseline; T2: week 4
Chen et al (2020)^32^	Robot	Taiwan, year not reported	20	Four LTC facilities	Mean age: 81·1 years; female: 65%	Pre-post	Intervention: social robot (PARO)	24/7 for 8 weeks	Depression (GDS); loneliness (UCLA loneliness scale); quality of life (WHO-QOL-OLD); cognition (MMSE); qualitative	T1: baseline; T2: 24 hours; T3: week 4; T4: week 8
D'Onofrio et al (2019)^33^	Robot	Ireland, Italy, UK, year not reported	38	Residential care, hospital and community, participants with dementia	Mean age: 77·1 years; female: 63·2%	Pre-post	Intervention: social robot (MARIO)	45 min, five times	Depression (CSDD); quality of life (QOL-AD); social support (MSPSS)	T1: baseline; T2: not reported
Fields et al (2021)^34^	Robot	USA, year not reported	15	Two LTC facilities	Mean age: 85·8 years; female: 73·3%	Pilot study	Intervention: social robot (NAO)	10 min, three times	Loneliness (UCLA loneliness scale); depression (GDS); mood (face scale)	T1: baseline; T2: after 3 sessions
Koh et al (2018)^35^	Robot	South Korea, 2016	33	LTC facility	Mean age: 86·5 years; female: 97%	Non-equivalent control pre-post	Intervention: social robot (PARO)	30 min, twice a week for 6 weeks	Cognitive function (MMSE); emotion (AER); problem behaviours (K-CMAI); social interactions	T1: baseline; T2: week 6
Lane et al (2016)^36^	Robot	USA, 2012–13	23	LTC facility, participants with dementia	Mean age: 80 years; female: 0%	Pre-post	Intervention: social robot (PARO)	No set time (on average, participants made 4·3 interactions apiece of minimum 5 min each)	Negative behavioural states; positive behavioural states	T1: 1 hour before intervention; T2: during intervention; T3: 1–2 hours post-intervention
Lazarou et al (2016)^37^	Environmental and wearable sensors	Greece, 2015	4	LTC facility, participants with dementia	Age: ≥65 years; female: 75%	Pre-post	Intervention: smart home environment	3–4 month period	Cognition (MMSE and MoCA); depression (HDRS); sleep; qualitative	T1: baseline; T2: month 4
Merilahti et al (2009)^38^	Environmental and wearable sensors	Finland, 2006	19	Assisted living facility	Mean age: 78 years; female: 73·7%	Feasibility trial	Intervention: environmentally embedded and wearable sensors	84 days on average	Acceptability; information collected	T1: baseline; T2: after trial
Mihailidis et al (2008)^39^	Environmental sensors	Canada, year not reported	8	LTC facility	Mean age: 85 years; female: 83·8%	Quasi-experimental time series ABAB	Intervention: environmentally embedded sensors (COACH†)	One session per day for 8 weeks	Handwashing; interactions with caregivers; function with independence	T1: baseline; T2: day 11; T3: day 21; T4: day 32; T5: day 42
Obayashi et al (2020)^40^	Robot and environmental sensors	Japan, 2015	2 participants, 4 caregivers	Nursing home	Mean age: 95·5 years; female: 100%	Feasibility study	Intervention: Sota robot plus sleep sensor (Nemuri SCAN‡)	4 days	Behavioural motivations; caregiver burden	T1: baseline; T2: day 2; T3: day 3; T4: day 4
Robinson et al (2013)^41^	Robot	New Zealand, year not reported	10 residents, 10 family members	Dementia unit	Age range: 71–93 years; female: 50%	Pilot study	Intervention: pet robot (PARO and Guide)	1 hour session	Acceptability	T1: after intervention
Robinson et al (2015)^42^	Robot	New Zealand, year not reported	21	LTC facility	Mean age: 84·9 years; female: 67%	Pilot study	Intervention: pet robot (PARO)	10 min	Blood pressure; heart rate	T1: baseline; T2: 10 min; T3: 15 min
Sung et al (2015)^43^	Robot	Taiwan, year not reported	12	LTC facility	Mean age: 77·2 years; female: 25%	Pilot study	Intervention: pet robot (PARO)	30 min, twice a week for 4 weeks	Communication/social skills (ACIS); activity participation (APS)	T1: baseline; T2: week 4

ABMI=Agitation Behavior Mapping Instrument. ACE=Addenbrooke's Cognitive Examination. ACIS=Assessment of Communication and Interaction Skills. ADLs=activities of daily living. AER=Apparent Emotion Rating scale. AES=Apathy Evaluation Scale. AI=Apathy Inventory. APADEM-NH=Apathy in Dementia, Nursing Home version. APS=Activity Participation Scale. AWS=Algase Wandering Scale. BARS=Brief Agitation Rating Scale. CMAI-SF=Cohen-Mansfield Agitation Inventory (short form). CSDD=Cornell Scale for Depression in Dementia. GDS=Geriatric Depression Scale. GIP-28=short version of the Dutch Behavioral Rating Scale for Geriatric Inpatients. HDRS=Hamilton Depression Rating Scale. IADLs=instrumental activities of daily living. K-CMAI=Korean version of the Cohen-Mansfield Agitation Inventory. LMBS=Lawton's Modified Behavior Stream. LTC=long-term care. MLAPS= modified Lexington Attachment to Pets Scale. MMSE=Mini-Mental State Examination. MoCA=Montreal Cognitive Assessment. MSPSS=Multidimensional Scale of Perceived Social Support. NPI-Q=Neuropsychiatric Inventory Questionnaire. OERS=Observed Emotion Rating Scale. PAINAD=Pain Assessment in Advanced Dementia Scale. QoL=AD=Quality of Life in Alzheimer's Disease. QUALID=Quality of Life in Late-stage Dementia. RAID=Rating Anxiety In Dementia. RCT=randomised controlled trial. T=timepoint. WHO-QOL-OLD=World Health Organization Quality of Life—older adults module.

* GAITRite: CIR Systems; Franklin, NJ, USA.

† COACH (Cognitive Orthosis for Assisting with aCtivites in the Home): Intelligent Assistive Technology and Systems Lab; Toronto, Canada.

‡ Nemuri SCAN: Paramount Bed; Tokyo, Japan.

Most studies had a small sample size, ranging from four to 490 (mean 70·8; SD 111·4). All studies were conducted with older people receiving LTC from either nursing homes, assisted living facilities, or dementia units. Only one study involved home-based LTC.

The interventions were of three broad types: AI-enhanced robots (n=24),^13^, ^14^, ^16^, ^17^, ^18^, ^19^, ^20^, ^21^, ^22^, ^23^, ^26^, ^27^, ^28^, ^30^, ^31^, ^32^, ^33^, ^34^, ^35^, ^36^, ^40^, ^41^, ^42^, ^43^ environmental sensors (n=6),^24^, ^25^, ^37^, ^38^, ^39^, ^40^ and wearable sensors (n=5).^15^, ^29^, ^37^, ^38^, ^40^ Three studies used a combination of sensor types.^37^, ^38^, ^40^ Intervention duration varied but regular sessions across several weeks were typical, with some studies delivering the intervention for up to a year. Few studies included one short session. Robots included in the studies were: AIBO (Sony; Tokyo, Japan), Cafero (Yujin Robot; Incheon, South Korea), Guide (ED Corporation; Seongnam, South Korea), MARIO (the MARIO Project, multiple collaborators), NAO (SoftBank Robotics; Tokyo, Japan), NeCoRo (Omron; Kyoto, Japan), PARO (Intelligent System; Toyama, Japan, and AIST; Tokyo, Japan), and Sota (VStone; Osaka, Japan, and NTT; Tokyo, Japan). Most robots provided companionship (eg, PARO, AIBO). Some robots monitored vital signs and delivered entertainment, video-calling, and cognitive games (eg, Guide, Cafero). Environmental sensors coached activities of daily living (eg, by wall-mounted sensors) and monitored health outcomes (eg, using under-mattress sensors, infrared motion [via passive infrared sensors], and gait sensors) in assisted living facilities.^24^, ^25^, ^37^, ^38^, ^39^, ^40^ Wrist-worn sensors monitored falls and health status in assisted living facilities and home-based LTC.^15^, ^29^, ^37^, ^38^, ^40^ Combined systems using wrist-worn and environmental sensors (eg, cameras, wireless tags) monitored health and functional status in assisted living facilities.^37^, ^38^, ^40^

Many outcomes were investigated, including depression (n=12),^14^, ^16^, ^20^, ^23^, ^24^, ^25^, ^26^, ^30^, ^32^, ^33^, ^34^, ^37^ quality of life (n=8),^14^, ^17^, ^20^, ^26^, ^28^, ^30^, ^32^, ^33^ agitation (n=5),^16^, ^18^, ^19^, ^20^, ^23^ acceptability (n=5),^14^, ^15^, ^30^, ^38^, ^41^ social interaction (n=5),^18^, ^27^, ^33^, ^35^, ^43^ cognition (n=5),^24^, ^25^, ^28^, ^35^, ^37^ loneliness (n=4),^13^, ^26^, ^32^, ^34^ behavioural states (n=3),^35^, ^36^, ^40^ anxiety (n=3),^19^, ^20^, ^23^ engagement and activity participation (n=3),^21^, ^22^, ^43^ sleep quality (n=3),^22^, ^23^, ^37^ mood (n=3),^22^, ^31^, ^34^ emotions (n=2),^20^, ^35^ apathy (n=2),^20^, ^28^ dependence (n=3),^14^, ^24^, ^39^ hospitalisation (n=2),^24^, ^29^ blood pressure (n=2),^18^, ^42^ heart rate (n=2),^18^, ^42^ neuropsychiatric symptomatology (n=2),^9^, ^28^ motor activity (n=2),^19^, ^23^ gait (n=2),^24^, ^25^ short physical performance battery (n=1),^24^ activities of daily living (n=1),^24^ balance (n=1),^25^ falls (n=1),^29^ attachment (n=1),^40^ caregiver burden (n=1),^40^ hair cortisol (n=1),^18^ wandering (n=1),^20^ pain (n=1),^23^ and medication use (n=1).^18^ Most studies measured outcomes at least at two timepoints, with one typically after intervention completion.

A summary of the outcomes assessed in at least three studies is shown in table 2 for controlled trials and table 3 for non-controlled trials.

**Table 2:** Summary of quantitative results—controlled trials

	**Outcome measure**	**Intervention**	**Timepoints measured**	Difference between timepoints*	**Comparison with control**
**Depression**					
Robinson et al (2013)^26^	GDS	PARO	T0: baseline; T1: week 12	Intervention score T1/T0: −0·64 (3·89); control score T1/T0: 0·40 (2·56)	p=0·97
Moyle et al (2013)^20^	GDS	PARO	T0: baseline; T1: week 5	Intervention: score T1/T0: −0·67	p>0·05†
Broadbent et al (2016)^14^	GDS	Guide and Cafero	T0: baseline; T1: week 12	Intervention score T1/T0: −0·7; control score T1/T0: −0·4	p>0·05†
Rantz et al (2017)^24^	GDS	Environmentally embedded sensor system	T0: baseline; T1: month 4; T2: month 8; T3: month 12	Not reported	p>0·05†
Rantz et al (2012)^25^	GDS	Environmental sensors	T0: baseline; T1: month 4; T2: month 8; T3: month 12;	Not reported	p>0·05†
Pu et al (2020)^23^	CSDD	PARO	T0: baseline; T1: week 6	Intervention score T1/T0: −1·65 (5·72); control score T1/T0: 0·73 (5·44)	p=0·158
Jøranson et al (2015)^16^	CSDD	PARO	T0: baseline; T1: week 12; T2: week 25	Intervention score T1/T0: −1·1, T2/T0: −1·8; control score T1/T0: 1·2, T2/T0: 2·4	T1/T0: p=0·98; T2/T0: p=0·03
**Quality of life**					
Robinson et al (2013)^26^	QOL-AD (self-rated and carer-rated)	PARO	T0: baseline; T1: week 12	Self-rated intervention score T1/T0: −1·33 (5·77); self-rated control score T1/T0: −1·88 (4·27); staff-rated intervention score T1/T0: −5·71 (7·65); staff-rated control score T1/T0: −7·06 (8·36)	Self-rated: p=0·64; staff rated: p=0·29
Moyle et al (2013)^20^	QOL-AD	PARO	T0: baseline; T1: week 5	Intervention score T1/T0: 5·0	p<0·05†
Jøranson et al (2016)^17^	QUALID	PARO	T0: baseline; T1: week 12; T2: week 25	Intervention score T1/T0: −0·21, T2/T0: 0·20; control score T1/T0: 2·39, T2/T0: 3·56	T1/T0: p=0·12; T2/T0: p=0·117
Valentí Soler et al (2015)^28^	QUALID	PARO and NAO	T0: baseline; T1: 3 months	Intervention score T1/T0: 1·31; dog score T1/T0: −0·43; control score T1/T0: −2·80	Control *vs* PARO: p=0·044; control *vs* dog: p=0·10; PARO *vs* dog: p=0·55
Broadbent et al (2016)^14^	QOL-AD	Guide and Cafero	T0: baseline; T1: week 12	Self-rated intervention score T1/T0: −0·4; self-rated control score T1/T0: −1·6; staff-rated intervention score T1/T0: 1·8; staff-rated control score T1/T0: 2·7	Self-rated, F-test (1,42): 0·43, p>0·05†; staff-rated, F-test (1,52): 0·04, p>0·05†
**Agitation**					
Jøranson et al (2015)^16^	BARS	PARO	T0: baseline; T1: week 12; T2: week 24	Intervention score T1/T0: −3·6, T2/T0: −5·51; control score T1/T0: −2·3, T2/T0: −3·9	T1/T0: p=0·098; T2/T0: p=0·048
Moyle et al (2013)^20^	CMAI-SF	PARO	T0: baseline; T1: week 10; T2: week 15	Not reported	PARO *vs* plush toy: p=0·68; PARO *vs* usual care: p=0·34; plush toy *vs* usual care: p=0·72
Pu et al (2020)^23^	CMAI-SF	PARO	T0: baseline; T1: week 6	Intervention score T1/T0: −0·14 (7·05); control score T1/T0: 1·86 (10·62)	p=0·45
Liang et al (2017)^18^	CMAI-SF	PARO	T0: baseline; T1: week 6; T2: week 12	Intervention score T1/T0: −0·7, T2/T0: −0·1; control score T1/T0: −3·0, T2/T0: −0·9	p=0·549
Libin et al (2004)^19^	ABMI	NeCoRo (Robot cat)	T0: baseline; T1: after two sessions	Intervention score T1/T0: 5·1; control score T1/T0: 3·9	Not reported

ABMI=Agitation Behavior Mapping Instrument. BARS=Brief Agitation Rating Scale. CMAI-SF=Cohen-Mansfield Agitation Inventory (short form). CSDD=Cornell Scale for Depression in Dementia. GDS=Geriatric Depression Scale. QOL-AD=Quality of Life in Alzheimer's Disease. QUALID=Quality of Life in Late-stage Dementia. T=timepoint. WHO-QOL-OLD=World Health Organization Quality of Life—older adults module.

* In this column, numbers in parentheses are standard deviations. T1/0 denotes score at timepoint 1 minus score at timepoint 0, and T2/0 denotes score at timepoint 2 minus score at timepoint 0.

† Exact p value was not reported in the article.

**Table 3:** Summary of quantitative results—non-controlled trials

	**Outcome measure**	**Intervention**	**Timepoints measured**	Difference between timepoints*
**Depression**				
Fields et al (2021)^34^	GDS	NAO	T1: baseline; T2: after 3 sessions	No dementia: −1·75 (1·39); with dementia: −0·25 (0·50); p=0·02
D'Onofrio et al (2019)^33^	CSDD	MARIO	T1: baseline; T2: not reported	−2·91 (0·79); p=0·100
Barrett et al (2019)^30^	CSDD	MARIO	T1: baseline; T2: week 4	0 (1·70); p=0·80
Chen et al (2020)^32^	GDS-SF	PARO	T1: baseline; T2: 24 hours; T3: week 4; T4: week 8	T4/T2: −6·55 (2·35), p<0·001†; T3/T2: −5·45 (2·21), p<0·001†; T4/T3: −1·10 (1·83), p<0·015
Lazarou et al (2016)^37^	HDRS	Smart home environment	T1: baseline; T2: month 4	−6·75 (2·32); p=0·01
**Quality of life**				
D'Onofrio et al (2019)^33^	QOL-AD	MARIO	T1: baseline; T2: not reported	5·85 (7·06); p=0·08
Barrett et al (2019)^30^	QOL-AD	MARIO	T1: baseline; T2: week 4	0·12 (2·55); p=0·61
Chen et al (2020)^32^	WHO-QOL-OLD	PARO	T1: baseline; T2: 24 hours; T3: week 4; T4: week 8	T4/T2: d=0·57, p<0·001†; T3/T2: d=0·54, p=0·01; T4/T3: d=0·16, p=0·326‡

CSDD=Cornell Scale for Depression in Dementia. GDS=Geriatric Depression Scale. GDS-SF=GDS short form. HDRS=Hamilton Depression Rating Scale. QOL-AD=Quality of Life in Alzheimer's Disease. T=timepoint. WHO-QOL-OLD=World Health Organization Quality of Life—older adults module.

* In this column, numbers in parentheses are standard deviations. T4/T2 denotes score at timepoint 4 minus score at timepoint 2; the same principle applies to T3/T2 and T4/T3. The differences were not reported in the papers (apart from Fields et al and Chen et al), but were calculated from the provided numbers.

† Exact p value was not reported in the article.

‡ Values reported in this cell are Cohen's d scores.

### Reporting biases

**Figure 2:**
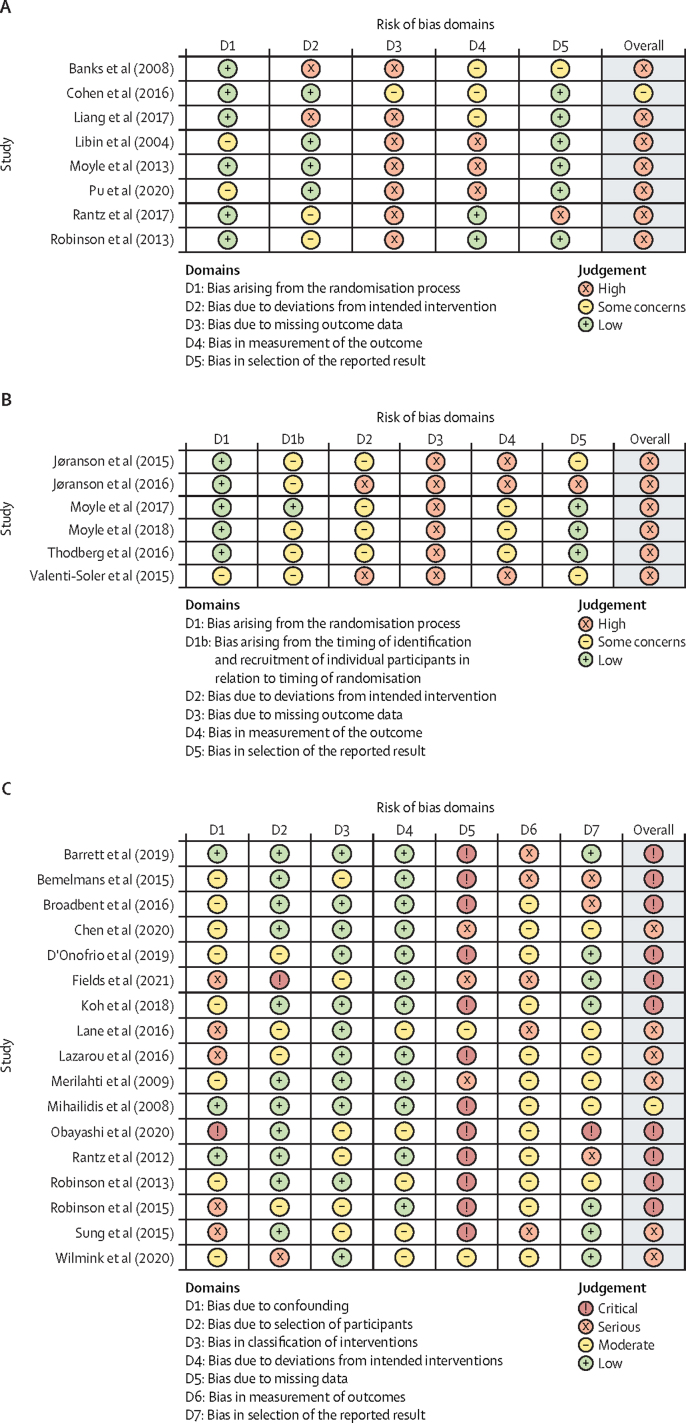
Risk of bias assessment (A) Revised Cochrane Risk of Bias tool for randomised trials (RoB 2). (B) Revised Cochrane Risk of Bias tool for cluster-randomised trials (RoB 2 CRT). (C) Risk of Bias in Non-Randomised Studies of Interventions (ROBINS-I).

The RoB 2 risk of bias tool was used to assess the quality of randomised trials. The majority of the included studies demonstrated high risk of bias, reducing confidence in the reported results (figure 2A). Domains assessing bias from the randomisation process and from selective reporting of results mostly showed low risk of bias, although some studies posed some concern. The most common concerns pertained to reporting missing outcome data and the appropriateness of outcome measures.

All studies assessed using the RoB 2 tool for cluster randomised trials showed high risk of bias (figure 2B). Concerns arose about the timing of participant identification and recruitment in relation to the timing of randomisation, and deviations from the intended intervention. Other issues pertained to the reporting of missing outcome data, outcome measurement, and selective reporting, with some studies failing to report data for all outcomes.

Few issues arose from the selection of participants, classification of the intervention, or deviations from the intended intervention in the non-controlled trials (ROBINS-I; figure 2C). However, there were concerns about the lack of reporting on potential confounders and bias arising from outcome measurement in several studies, reducing confidence in the reported results. No studies reported on how missing data were handled. Selective reporting of the outcomes appeared to be an issue in some studies.

### Acceptability

Three controlled trials and two non-controlled trials investigated the acceptability of different AI-enhanced interventions. Acceptability of social robots was mixed, and varied by robot and use case (eg, clinical *vs* entertainment). Acceptability was poor for environmental and wearable sensors in two studies, although in one study the feedback varied between caregivers and LTC consumers.^15^

Of three controlled trials, two evaluated social robots. Rest-home and hospital residents provided mixed feedback on robots Cafero and Guide after a 12-week intervention.^14^ In a dementia unit, PARO was well received and favoured over Guide.^41^ A third controlled trial reported poor acceptability of an environmentally embedded movement monitoring system among residents, who questioned its usefulness.^15^ However, acceptability was higher in caregivers, who liked its ease of use and ability to ensure the safety of older people.

Of the two non-controlled trials, one reported that the social robot MARIO (which aimed to reduce loneliness) was acceptable to the majority of participants but there were concerns about people with dementia's awareness of the robot speaking, and issues with appearance and accessibility.^30^ Another non-controlled trial investigated a package of health monitoring sensors, which were criticised and poorly received by participants.^38^ Users reported that the wearables were uncomfortable, the bed sensors interrupted sleep, and they felt disturbed by constant monitoring. Other studies reported on outcomes that might contribute positively to acceptability, such as the forming of attachments and reduced caregiver burden.^13^, ^40^

### Depression symptoms

Six controlled trials tested the effectiveness of AI-enhanced robots on depressive symptoms. Five studies did not find significant differences between the intervention and control group. Liang and colleagues found an improvement in depressive symptoms from baseline in both the PARO group and control group in a small study with people living with dementia.^18^ However, this effect was only present after 6 weeks, and depressive symptoms increased in the PARO group but not in the control group after 12 weeks. Two studies evaluating environmental sensors did not find any significant differences between the intervention and control group on depressive symptoms.^24^, ^25^ Five non-controlled trials testing robots and smart home environments found some improvement in depressive symptoms when comparing baseline with follow-up (table 3).

### Quality of life

Five controlled trials assessed the effect of AI-enhanced robots on quality of life, and only two found a significant effect (table 2). Both studies assessed the effect of PARO. Moyle and colleagues used a cross-over design, exposing recipients to PARO for 5 weeks with a 3-week washout period. PARO was associated with a positive moderate influence on the Quality of Life in Alzheimer's Disease (QOL-AD) scale.^20^ The second study compared PARO with a standard care control and a real trained dog in a nursing home.^28^ Exposure occurred 2 days a week for 3 months. Statistically significant differences were found in scores on the Quality of Life in Late-Stage Dementia (QUALID) scale, with the PARO group decreasing in quality of life in comparison to the control. Three non-controlled trials assessed the impact of social robots on quality of life and found mixed results, with only one study finding a positive effect on quality of life (table 3).

### Agitation

Five controlled trials investigated the effect of pet robots on agitation and reported mixed results. Three studies found small but significant decreases in agitation while two studies did not find significant differences between the intervention and control group.^16^, ^18^, ^19^, ^21^, ^23^

### Social outcomes

Two controlled trials and two non-controlled trials evaluated the effect of social robots on loneliness. AIBO and PARO were found to significantly decrease loneliness in controlled trials.^13^, ^26^ PARO and NAO were shown to decrease loneliness in non-controlled trials.^32^, ^34^

Five studies assessed social participation. A controlled trial using PARO reported significant improvement in participant communication with nursing home staff.^18^ Two non-controlled trials reported a significant improvement in communication and social skills after PARO interventions.^35^, ^43^ The robot MARIO was also shown to enhance perceptions of social support within participants.^33^

### Behavioural outcomes

Two controlled trials and one non-controlled trial demonstrated that participants who interacted with PARO significantly improved overall activity participation and were more verbally, physically, and visually engaged.^21^, ^27^, ^43^

One controlled and three non-controlled trials assessed behavioural states, with studies indicating observed decrease in negative behaviour^36^ and improved behavioural motivation.^40^ A controlled trial found that exposure to PARO was associated with increased levels of wandering (a behaviour associated with disease progression that can leave people lost or confused) in people living with dementia,^20^ while another study found a significant decrease in problem behaviour scores in this population.^35^

Three studies investigated independence. No significant changes were found in participants’ degree of dependency after using Guide or Cafero robots.^14^ However, the degree to which environmental sensors can aid in independence and affect daily living is mixed.^24^, ^39^

A controlled study of an environmentally embedded sensor system showed no significant differences in hospitalisation rates.^24^ However CarePredict (CarePredict; Plantation, FL, USA), a wearable sensor, was associated with lower hospitalisation rates in older people.^29^

### Neuropsychiatric and cognitive outcomes

Five studies evaluated cognitive function. Environmental sensor systems were found to have no significant effect on cognitive function in two controlled trials.^24^, ^25^ However, a non-controlled trial demonstrated significant improvement in cognitive function.^37^ Some authors report no significant effect of PARO on cognition^28^, ^35^ or neuropsychiatric symptoms,^18^ while others suggest PARO is associated with an improvement in night-time behavioural disturbances.^28^

### Physical capacity outcomes

Motor activity was assessed in two controlled trials using PARO. One study found no significant effect,^23^ while another found it aided in reducing motor activity.^19^ Gait parameters and physical performance were shown to improve in two controlled trials of environmental sensor systems, although no improvements in balance were found.^24^, ^25^ A wearable sensor was not found to have any significant effect on falls.^29^

### Psychological outcomes

Mixed results were found for the effect of social robots on anxiety. One study reported a small decrease in anxiety following exposure to PARO,^20^ while others report no significant effect.^23^ NeCoRo showed no significant effect on anxiety.^19^ Mood and emotion were shown to improve in four trials using PARO,^20^, ^22^, ^31^, ^35^ and in one trial using NAO.^34^ No significant effects were found on levels of apathy following a PARO intervention.^20^ However, apathy significantly decreased in a study using NAO.^28^

### Other health outcomes

Two controlled trials investigated the effect of PARO on sleep quality and found mixed results. One study showed a significant increase in daytime wakefulness and reduction of daytime sleep,^23^ whereas another study showed no evidence of improvement in sleeping patterns.^22^ A non-controlled trial of an environmental and wearable sensor system reported a significant improvement in sleep duration.^37^

The results regarding blood pressure and heart rate are mixed. One long-term controlled trial reported no significant differences between the participants in the PARO intervention group and the control group on these outcomes.^18^ By contrast, in a non-controlled trial, PARO was associated with a significant decrease in blood pressure and heart rate over a short period.^42^

PARO was not found to have any significant effect on pain,^23^ cortisol,^18^ or medication use.^18^

## Discussion

### Summary and context

This review found that although the acceptability of AI-enhanced interventions in LTC services was mixed, few conclusions can be drawn on the effectiveness of those interventions among older people. Some evidence was found in support of social robots for improving social participation and mood; however, the results were mixed and less conclusive regarding the other technologies and outcomes. Most studies assessed diverse outcomes, which were difficult to synthesise in a meaningful manner and made a meta-analysis impossible. Moreover, many studies were underpowered with a moderate to critical risk of bias.

### Contextualisation

This review is the first to investigate the acceptability and effectiveness of AI-enhanced interventions for older people receiving LTC. The results align with previous reviews which demonstrate the acceptability and preliminary effectiveness of AI-enhanced robots for improving psychosocial outcomes of older people receiving LTC services.^44^, ^45^

Systematic reviews of AI-enhanced intervention research in other health-care contexts have reported similar quality concerns to this review. These have included poor reporting,^46^ small sample sizes,^47^ a lack of external validation of results,^46^, ^48^ issues in the blinding of outcome assessments, and incomplete outcome data.^44^, ^45^ However, issues in the field might explain these methodological limitations. The high cost and low availability of robots makes it difficult to recruit large samples. Moreover, the prohibitive cost of robots might explain the lack of research representation from low-income and middle-income countries. Another challenge is that there are often technical reliability issues, meaning that work is required to maintain the devices during studies. Many robots are prototypes and are not as robust as commercially available technologies like mobile phones. It is also difficult to get follow-up data from older people in LTC settings for self-report questionnaires due to illness, fatigue, and mortality. Research in an LTC context might require a more pragmatic approach to conduct studies in the field.

### Limitations

This review explored the acceptability and effectiveness of AI-enhanced interventions with older people receiving LTC services. This, however, meant that the outcomes, settings, types of interventions, and study designs varied drastically, making comparison difficult. Acceptability can vary substantially depending on the technology design, use case (eg, whether it addresses an unmet clinical need), and its degree of invasiveness. Moreover, both acceptability and effectiveness might be impacted by the LTC context in which the research took place (eg, a dementia unit *vs* home-based LTC). The studies included in the review had high risk of bias in many domains, with issues particularly around missing data and selective outcome reporting. This poses an issue when trying to reach valuable conclusions about AI on outcomes, especially when underpowered. Follow-up times were on average relatively short, which made it difficult to assess the long-term impact and acceptability of the interventions. The findings of the studies are also difficult to generalise beyond the settings they were carried out in, with no studies conducted in low-income and middle-income settings. This might be partially attributable to the high cost of AI-enhanced interventions, which might hamper uptake in lower-income settings. We found that there is promising, but still inconclusive literature on AI-enhanced social robots used by older people using LTC services. In contrast, very few studies have been conducted with AI-enhanced smart technologies (eg, environmental and wearable sensors), and pragmatic clinical trials are needed to mature the field of research on the acceptability and effectiveness of AI-enhanced technologies.

This review had several limitations which might have affected the findings. Although the search results were not restricted by language, the searches were conducted using English search terms in English language databases. This might have limited the foreign language results to articles that included English abstracts or keywords. Indeed, several foreign language results were found. However, additional literature could have been identified from foreign language databases with translated search terms. Moreover, Web of Science, an interdisciplinary database that covers IEEE Xplore, was used to search computer science and engineering literature. A search involving additional computer science databases might have uncovered more results, albeit less likely clinical trials.

### Implications

#### Ethical Implications

Several studies have reported ethical considerations for AI-enhanced interventions as part of LTC services.^6^, ^49^ Older people living with dementia might be deceived into believing the robot is a real pet,^49^ and might be at risk of infantilisation from some technologies. Some older people form attachments with robots and experience distress when separated at the end of a trial.^50^ However, the severity and chronicity of separation distress with robots is understudied, and it remains unclear what strategies are appropriate for ending older peoples’ relationships with robots.

AI-enhanced sensors might contribute to depersonalisation of care and prompt surveillance concerns in LTC consumers.^6^ Indeed, concerns about surveillance and data confidentiality were reported in several of the included studies. In some studies, concerns were alleviated over time when trust in the system developed. However, clear communication about data protection and privacy processes to LTC consumers, caregivers, and facility staff should be of concern in future work.^51^ Robust data protection safeguards are of paramount importance to the acceptability and uptake of these technologies in LTC services.

Older people can have varied responses to social robots.^6^, ^14^ Some older people have been shown to have good engagement with robots, whereas others have been disinterested or responded negatively, perhaps due to higher baseline agitation.^14^ However, acceptability was found to improve over time in some studies.^45^ There is some reporting of possessiveness towards social robots, specifically PARO, which must be considered when sharing the robot.^6^ These findings have implications for practice, suggesting that consideration of individual needs and an implementation strategy are necessary to protect against unanticipated harms.

Studies evaluating sensors have raised concerns regarding the timing of equipment installation, which requires greater consideration in future work. Specifically, trials involving people with dementia report that installation should take place prior to more severe dementia onset and progression.^51^, ^52^

#### Future research and technology development

This review provides direction for future research and development of AI-enhanced interventions in LTC services. Many forms of AI-enhanced interventions (eg, conversational agents, smartphone applications) have yet to be tested through clinical trials with older people receiving LTC. It is possible that computer or smartphone-based interventions might be less expensive and more scalable methods of supporting LTC services. Indeed, Amazon's Alexa could support independent living, wellbeing, and connecting LTC users with resources and other people, although privacy concerns would need to be addressed.^53^ Screen-based, AI-enhanced interventions can present unique advantages over robots in terms of accessibility, including in resource-limited settings, and they should be evaluated in future trials. Moreover, AI-enhanced interventions should be tested in LTC services in low-income and middle-income countries as no trials have been conducted to date.

This review found that the scope of LTC services were not adequately covered by AI-enhanced interventions. Most interventions focused on social participation, mental capacity, or psychosocial outcomes. More interventions are needed that focus on assisting older people with activities of daily living and managing chronic health conditions. The review identified several environmental and wearable sensors that incorporated AI techniques for detecting outcomes such as frailty, fractures, abnormal daily activities in people living with dementia, disease exacerbations, falls, neuropsychiatric symptoms, and changes in health status. However, the technologies were in the development phase and had yet to be externally validated in LTC services. Increasing funding to support research on AI-enhanced interventions in LTC services, along with integrative collaborations between health and computing experts that ensure scientific rigour, may help to address the challenges of research in this field.

## Conclusions

The large heterogeneity of study designs, sample sizes, role of the technology, outcome measurement, and reporting make it difficult to reach conclusions on whether AI-enhanced interventions for LTC services are of significant benefit and should be considered for evidence-based recommendation in the future. Although some promising results were found, further research is needed. Outcomes for AI-enhanced intervention research should be standardised, ideally with a prioritisation exercise so that evidence is more easily comparable. Future research should adhere to the Consolidated Standards of Reporting Trials–Artificial Intelligence (CONSORT-AI)^54^ extension to reduce reporting biases. It is paramount that solutions included in future studies are the most appropriate for the needs of older people receiving LTC and to acknowledge that in some cases not all individuals will benefit from these technologies. Until then, AI-enhanced interventions could be considered as part of a technology development race, as opposed to being effective and acceptable solutions for LTC delivery.

## Declaration of interests

Soul Machines (a New Zealand-based AI company) supported KL with a PhD stipend at the time of the research and currently employs her as a Postdoctoral Research Associate (but not at the time the research was conducted); Soul Machines also contracts EB for consultancy work. Soul Machines had no say in the conduct of the study, its interpretation, and its conclusions. All other authors declare no competing interests. The views expressed in this paper are those of the authors and do not necessarily reflect the views of WHO.
