# Supplementary material for: Artificial intelligence for older people receiving long-term care: a systematic review of acceptability and effectiveness studies
Source: Lancet Healthy Longev. 2022 Apr;3(4):e286–97. doi: 10.1016/S2666-7568(22)00034-4 (PMC8979827; doi:10.1016/S2666-7568(22)00034-4)
Supplement: Supplementary appendix [file mmc1.pdf]

THE LANCET
Healthy Longevity

Supplementary appendix

This appendix formed part of the original submission and has been peer reviewed. We post it as supplied by the authors.

Supplement to: Loveys K, Prina M, Axford C, et al. Artificial intelligence for older people receiving long-term care: a systematic review of acceptability and effectiveness studies. *Lancet Healthy Longev* 2022; **3:** e286–97.

Appendix 1: PRISMA CHECKLIST

Section and Topic	Item #	Checklist item	Location where item is reported
**TITLE**			
Title	1	Identify the report as a systematic review.	1
**ABSTRACT**			
Abstract	2	See the PRISMA 2020 for Abstracts checklist.	2
**INTRODUCTION**			
Rationale	3	Describe the rationale for the review in the context of existing knowledge.	4, 7-8
Objectives	4	Provide an explicit statement of the objective(s) or question(s) the review addresses.	8
**METHODS**			
Eligibility criteria	5	Specify the inclusion and exclusion criteria for the review and how studies were grouped for the syntheses.	9, Appendix pgs. 8-10
Information sources	6	Specify all databases, registers, websites, organisations, reference lists and other sources searched or consulted to identify studies. Specify the date when each source was last searched or consulted.	9
Search strategy	7	Present the full search strategies for all databases, registers and websites, including any filters and limits used.	Appendix pgs. 4-7.
Selection process	8	Specify the methods used to decide whether a study met the inclusion criteria of the review, including how many reviewers screened each record and each report retrieved, whether they worked independently, and if applicable, details of automation tools used in the process.	9
Data collection process	9	Specify the methods used to collect data from reports, including how many reviewers collected data from each report, whether they worked independently, any processes for obtaining or confirming data from study investigators, and if applicable, details of automation tools used in the process.	10
Data items	10a	List and define all outcomes for which data were sought. Specify whether all results that were compatible with each outcome domain in each study were sought (e.g. for all measures, time points, analyses), and if not, the methods used to decide which results to collect.	10
	10b	List and define all other variables for which data were sought (e.g. participant and intervention characteristics, funding sources). Describe any assumptions made about any missing or unclear information.	10
Study risk of bias assessment	11	Specify the methods used to assess risk of bias in the included studies, including details of the tool(s) used, how many reviewers assessed each study and whether they worked independently, and if applicable, details of automation tools used in the process.	10
Effect measures	12	Specify for each outcome the effect measure(s) (e.g. risk ratio, mean difference) used in the synthesis or presentation of results.	10
Synthesis methods	13a	Describe the processes used to decide which studies were eligible for each synthesis (e.g. tabulating the study intervention characteristics and comparing against the planned groups for each synthesis (item #5)).	10
	13b	Describe any methods required to prepare the data for presentation or synthesis, such as handling of missing summary statistics, or data conversions.	10

Section and Topic	Item #	Checklist item	Location where item is reported
	13c	Describe any methods used to tabulate or visually display results of individual studies and syntheses.	10
	13d	Describe any methods used to synthesize results and provide a rationale for the choice(s). If meta-analysis was performed, describe the model(s), method(s) to identify the presence and extent of statistical heterogeneity, and software package(s) used.	10
	13e	Describe any methods used to explore possible causes of heterogeneity among study results (e.g. subgroup analysis, meta-regression).	n/a
	13f	Describe any sensitivity analyses conducted to assess robustness of the synthesized results.	n/a
Reporting bias assessment	14	Describe any methods used to assess risk of bias due to missing results in a synthesis (arising from reporting biases).	10
Certainty assessment	15	Describe any methods used to assess certainty (or confidence) in the body of evidence for an outcome.	10
**RESULTS**			
Study selection	16a	Describe the results of the search and selection process, from the number of records identified in the search to the number of studies included in the review, ideally using a flow diagram.	10, Figure 1
	16b	Cite studies that might appear to meet the inclusion criteria, but which were excluded, and explain why they were excluded.	10, 21
Study characteristics	17	Cite each included study and present its characteristics.	10-11,Table 1, Table 2, Table 3
Risk of bias in studies	18	Present assessments of risk of bias for each included study.	16-17, Figure 2
Results of individual studies	19	For all outcomes, present, for each study: (a) summary statistics for each group (where appropriate) and (b) an effect estimate and its precision (e.g. confidence/credible interval), ideally using structured tables or plots.	Table 2, Table 3
Results of syntheses	20a	For each synthesis, briefly summarise the characteristics and risk of bias among contributing studies.	16-17
	20b	Present results of all statistical syntheses conducted. If meta-analysis was done, present for each the summary estimate and its precision (e.g. confidence/credible interval) and measures of statistical heterogeneity. If comparing groups, describe the direction of the effect.	12-16
	20c	Present results of all investigations of possible causes of heterogeneity among study results.	12-16
	20d	Present results of all sensitivity analyses conducted to assess the robustness of the synthesized results.	n/a
Reporting biases	21	Present assessments of risk of bias due to missing results (arising from reporting biases) for each synthesis assessed.	16-17
Certainty of evidence	22	Present assessments of certainty (or confidence) in the body of evidence for each outcome assessed.	16-17
**DISCUSSION**			
Discussion	23a	Provide a general interpretation of the results in the context of other evidence.	17-18
	23b	Discuss any limitations of the evidence included in the review.	18-19

Section and Topic	Item #	Checklist item	Location where item is reported
	23c	Discuss any limitations of the review processes used.	18-19
	23d	Discuss implications of the results for practice, policy, and future research.	19-21
**OTHER INFORMATION**			
Registration and protocol	24a	Provide registration information for the review, including register name and registration number, or state that the review was not registered.	9
	24b	Indicate where the review protocol can be accessed, or state that a protocol was not prepared.	9
	24c	Describe and explain any amendments to information provided at registration or in the protocol.	9
Support	25	Describe sources of financial or non-financial support for the review, and the role of the funders or sponsors in the review.	3
Competing interests	26	Declare any competing interests of review authors.	22
Availability of data, code and other materials	27	Report which of the following are publicly available and where they can be found: template data collection forms; data extracted from included studies; data used for all analyses; analytic code; any other materials used in the review.	10

From: Page MJ, McKenzie JE, Bossuyt PM, Boutron I, Hoffmann TC, Mulrow CD, et al. The PRISMA 2020 statement: an updated guideline for reporting systematic reviews. BMJ 2021;372:n71. doi: 10.1136/bmj.n71

Appendix 2: Search Strategy

EMBASE, Ovid, Global Health, PsycINFO

1. "older adult*" OR elderly OR "older person*" OR senior* OR aged

2. "nursing home" OR "care home" OR "residential home" OR "extended care" OR "longterm care" OR "long-term care" OR "assisted living" OR "residential care" OR caregiver OR "social care" OR "home care" OR "home-based care" OR "respite care"

3. "artificial intelligence" OR "machine learning" OR "support vector machine" OR "neural networks" OR "vector machine" OR "machine learning" OR "deep learning" OR "ensemble learning" OR "deep network" OR "convolutional network" OR "neural network" OR "bayesian network" OR "classification tree" OR "regression tree" OR "probability tree" OR "nearest neighbor" OR "nearest neighbour" OR "fuzzy logit" OR "fuzzy logic" OR "fuzzy logistic" OR "native bayes" OR "genetic algorithm*" OR "multilayer perception" OR "random forest" OR lasso* OR kernel* OR "elastic net*" OR fuzzy OR “classification algorithm” OR “artificial neural network” OR automated OR algorithm OR wearable* OR sensor* OR “internet of things” OR robot* OR “digital human*” OR “virtual agent*” OR “conversation* agent” OR chatbot OR app

4. "randomized controlled trial" OR "controlled clinical trial" OR "pragmatic clinical trial" OR "equivalence trial" OR "clinical trial" OR randomization OR "random allocation" OR "double-blind method" OR "double blind procedure" OR "double-blind studies" OR "single-blind method" OR "single blind procedure" OR "single-blind studies" OR placebo* OR "control group*" OR random* OR sham OR "singl* blind*" OR "singl* dumm*" OR "singl* mask*" OR "doubl* blind*" OR "doubl* dumm*" OR "doubl* mask*" OR "tripl* blind*"

OR "tripl* dumm*" OR "tripl* mask*" OR "trebl* blind*" OR "trebl* dumm*" OR "trebl* mask*" OR "control* study" OR "control* studies" OR "control* group*" OR "control* trial*" OR nonrandom* OR non random* OR non-random* OR quasi-random* OR quasirandom* OR allocated OR "open label study" OR "open label studies" OR "open label trial*" OR "open-label study" OR "open-label studies" OR "open-label trial" OR "phase 3" OR "phase III" OR "equivalence study" OR "equivalence studies" OR "equivalence trial*" OR "superiority study" OR "superiority studies" OR "superiority trial*" OR "non-inferiority study" OR "non-inferority studies" OR "non-inferiority trial*" OR "noninferiority study" OR "noninferiority studies" OR "noninferiority trial*" OR "pragmatic study" OR "pragmatic studies" OR "pragmatic trial*" OR "practical trial*" OR "quasiexperimental study" OR "quasiexperimental studies" OR "quasiexperimental trial*" OR "quasi-experimental study" OR "quasi-experimental studies" OR "quasiexperimental trial*"

5. 1 and 2 and 3 and 4

6. Remove duplicates from 5

Web of Science

("older adult*" OR elderly OR "older person*" OR senior* OR aged) AND ("nursing home" OR "care home" OR "residential home" OR "extended care" OR "longterm care" OR "long-term care" OR "assisted living" OR "residential care" OR caregiver OR "social care" OR "home care" OR "home-based care" OR "respite care") AND ("artificial intelligence" OR "machine learning" OR "support vector machine" OR "neural networks" OR "vector machine" OR "machine learning" OR "deep learning" OR "ensemble learning" OR "deep network" OR "convolutional network" OR "neural network" OR "bayesian network" OR "classification tree" OR "regression tree" OR "probability tree" OR "nearest neighbor" OR "nearest neighbour" OR "fuzzy logit" OR "fuzzy logic" OR "fuzzy logistic" OR "native bayes" OR "genetic algorithm*" OR "multilayer perception" OR "random forest" OR lasso* OR kernel* OR "elastic net*" OR fuzzy OR “classification algorithm” OR “artificial neural network” OR automated OR algorithm OR wearable* OR sensor* OR “internet of things” OR robot* OR “digital human*” OR “virtual agent*” OR “conversation* agent” OR chatbot OR app) AND ("randomized controlled trial" OR "controlled clinical trial" OR "pragmatic clinical trial" OR "equivalence trial" OR "clinical trial" OR randomization OR "random allocation" OR "double-blind method" OR "double blind procedure" OR "double-blind studies" OR "single-blind method" OR "single blind procedure" OR "single-blind studies" OR placebo* OR "control group*" OR random* OR sham OR "singl* blind*" OR "singl* dumm*" OR "singl* mask*" OR "doubl* blind*" OR "doubl* dumm*" OR "doubl* mask*" OR "tripl* blind*" OR "tripl* dumm*" OR "tripl* mask*" OR "trebl* blind*" OR "trebl* dumm*" OR "trebl* mask*" OR "control* study" OR "control* studies" OR "control* group*" OR "control* trial*" OR nonrandom* OR non random* OR non-random* OR quasi-random* OR quasirandom* OR allocated OR "open label study" OR "open label studies" OR "open label trial*" OR "open-label study" OR "open-label studies" OR "open-label trial" OR

"phase 3" OR "phase III" OR "equivalence study" OR "equivalence studies" OR "equivalence trial*" OR "superiority study" OR "superiority studies" OR "superiority trial*" OR "non-inferiority study" OR "non-inferority studies" OR "non-inferiority trial*" OR "noninferiority study" OR "noninferiority studies" OR "noninferiority trial*" OR "pragmatic study" OR "pragmatic studies" OR "pragmatic trial*" OR "practical trial*" OR "quasiexperimental study" OR "quasiexperimental studies" OR "quasiexperimental trial*" OR "quasi-experimental study" OR "quasi-experimental studies" OR "quasiexperimental trial)

Appendix 3: Eligibility criteria

**Category**	**Inclusion criteria**	**Exclusion criteria**
Publication type	Peer-reviewed journal articles, refereed full-length conference papers	Theses, dissertations, other non- peer-reviewed literature
Study design	Randomised controlled trials, controlled (non-randomised) clinical trials, pre- and post-evaluation clinical trials, pilot studies, feasibility and acceptability studies	Opinion piece, protocol, laboratory experiments, qualitative studies, review articles
Participants	Older adults with a mean age of 65 years or older	Populations with a mean age of less than 65 years
Intervention	Technology that included AI techniques for healthcare monitoring and/or intervention delivery.<br><br>The technology could have focused on supporting physical capacity, mental capacity, physical health, or mental health outcomes.<br><br>AI techniques could have included machine learning or types of machine learning (e.g., neural networks, deep learning, reinforcement learning, ensemble learning, Bayesian networks, convolutional networks, classification trees, nearest neighbour, classification algorithms, random forest, support vector machines, fuzzy logic, genetic algorithms), patten detection, computer vision, natural language processing, process automation, robotics.	Studies were not excluded based on the technology's hardware form. However, the technology needed to use an AI technique to be included.
Comparator	Treatment as usual, a waitlist control, a different form of AI, an active control that does not	N/A

	include AI (e.g., an alternative technology, human delivery)	
Outcomes	Acceptability, or a health outcome pertaining to physical or mental capacity or health. Health outcomes could include independence, rehabilitation, mobility, vision, hearing, incontinence, falls, daily functioning, activities of daily living, instrumental activities of daily living, muscle strength, balance, chair stand, timed up and go, overall physical functioning, mental capacity, cognitive impairment, dementia symptoms, gait speed, grip strength, forced expiratory volume, symptom recovery, depression symptoms, anxiety symptoms, stress, loneliness, healthcare utilization, healthcare engagement.*	Studies were excluded that only reported on the sensitivity, specificity, or accuracy of the AI model and did not evaluate acceptability or the effect of the technology on a health outcome. Excluded studies could have also focused on outcomes that were not identified in the inclusion criteria (e.g., cost-effectiveness).
Setting	Long-term care facilities, community day care centres/facilities, or home-based long-term care.<br><br>Long-term care facilities could have included an assisted living facility, a nursing home, skilled nursing facilities, residential facilities, residential long-term care facilities, or respite care.**	Studies where the technology was not evaluated in a long-term care setting.
Other		Studies were not excluded based on methodological quality

* Outcomes were informed by the Integrated Care for Older People (ICOPE) guidelines.[1,2]

** The definition of long-term care and long-term care facilities were informed by the World Health Organization.[3,4] Long-term care services address the health, personal care, and social needs of individuals.

1. World Health Organization. Integrated care for older people (ICOPE) guidelines on community-level interventions to manage declines in intrinsic capacity evidence profile: malnutrition [Internet]. Switzerland (Geneva): World Health Organization; 2017 [Cited October 13 2021]. Available from: https://apps.who.int/iris/handle/10665/342251.
2. World Health Organization. Integrated care for older people (ICOPE) guidelines on community-level interventions to manage declines in intrinsic capacity evidence profile: mobility loss [Internet]. Switzerland (Geneva): World Health Organization; 2017 [Cited October 13 2021]. Available from: https://apps.who.int/iris/handle/10665/342253.
3. World Health Organization. Preventing and managing COVID-19 across long-term care services [Internet]. Switzerland (Geneva): World Health Organization; 2020 [Cited October 13 2021]. Available from: https://www.who.int/publications/i/item/WHO-2019-nCoV-Policy_Brief-Long-term_Care-2020.1.
4. World Health Organization. World report on ageing and health [Internet]. Switzerland (Geneva): World Health Organization; 2015 [Cited October 13 2021]. Available from: https://apps.who.int/iris/handle/10665/186463.
